# Impaired hypoxic ventilatory drive induced by diabetic autonomic neuropathy, a cause of misdiagnosed severe cardiac events: brief report of two cases

**DOI:** 10.1186/s12872-021-01944-4

**Published:** 2021-03-17

**Authors:** Louis Schubert, Suzanne Laroche, Agnès Hartemann, Olivier Bourron, Franck Phan

**Affiliations:** 1 Diabetology Department, Pitié-Salpêtrière-Charles Foix Hospital, AP-HP, 75013 Paris, France; 2 Sorbonne Université, Paris, France; 3 INSERM, UMR_S 1138, Centre de Recherche Des Cordeliers, Paris 06, France; 4 Institute of Cardiometabolism and Nutrition ICAN, Paris, France

**Keywords:** Respiratory failure, Cardiopulmonary resuscitation, Cardiac autonomic neuropathy, Hypoxia, Type 1 diabetes mellitus

## Abstract

**Background:**

Sudden cardiac deaths are twice more frequent in diabetic patients with cardiac autonomic neuropathy. Sudden cardiac death etiologies remain unclear and no recommendations are made to identify factors associated with cardiorespiratory arrest in diabetic patients. We hypothesized, from two clinical cases, that impaired hypoxic ventilatory drive, induced by diabetic autonomic neuropathy, is a cause of misdiagnosed severe cardiac events.

**Case presentation:**

We describe the cases of two patients with isolated low blood saturation on pulse oximeter during the systematic nurse check-up (77% and 85% respectively) contrasting with the absence of any complaint such as dyspnea, polypnea or other respiratory insufficiency signs observed during the clinical examination. Arterial blood gas measurements subsequently confirmed that blood oxygen saturation was low and both patients were indeed hypoxemic. Patient 1 suffered from vascular overload complicated by cardiac arrest caused by hypoxemia in light of the quick recovery observed after ventilation. Pulmonary edema was diagnosed in patient 2. The common denominator of these 2 cases described in this brief report is the absence of respiratory failure clinical signs contrasting with the presence of confirmed hypoxemia. Also, in both cases, such absence of precursory signs seems to be induced by an impaired ventilatory drive to hypoxemia. This appears to be related to the autonomic diabetic neuropathy encountered in those 2 patients.

**Conclusions:**

Therefore, we describe, in this brief report, cardiac autonomic neuropathy as a cause of impaired hypoxic ventilatory drive involved in severe acute cardiorespiratory events in two type 1 diabetic patients. We assume that altered response to hypoxemia due to cardiac autonomic neuropathy and non-functional central neurological breathing command could play a key role in sudden deaths among diabetic patients. An important point is that hypoxemia can be easily missed since no clinical signs of respiratory failure are reported in these two clinical cases. Systematic screening of cardiac autonomic neuropathy in diabetic patients and proactive detection of impaired hypoxic ventilatory drive for early management (e.g. treatment of hypoxemia) should be systematically undertaken in diabetic patients to prevent its dramatic consequences such as cardiorespiratory arrest and death.

## Background

Among diabetic complications, neuropathy has been thought to be nonlethal and is often poorly evaluated.. Different surveys reveal that about only 65% of diabetic patients yearly have a 10-g monofilament testing for neuropathy screening [1] and certainly many fewer have a cardiac autonomic neuropathy screening. The reported prevalence of cardiac autonomic neuropathy (CAN) varies greatly according to the criteria used to define it, as well as to the characteristics of the population investigated. CAN prevalence ranges from as low as 2.5% in the primary prevention cohort in the Diabetes Control and Complications Trial (DCCT) [2] to as high as 90% in patients with long-standing type 1 diabetes who were potential candidates for pancreas transplantation [3]. Screening for CAN among patients with diabetes represents a major issue for its prediction of syncope recurrence. In fact, diabetic CAN is associated with a higher rate of vaso-vagal syncope recurrence in a 12-month of follow-up study [4]. Furthermore, several other studies suggest that the mortality rate of diabetic patients is higher in those with established diabetic neuropathy, particularly CAN, than in those without [5]. Of note, while sudden cardiac deaths (SCD) mainly account for such increased mortality rate in diabetic patients suffering from CAN, SCD mechanisms are probably multiple and not yet clearly understood.

Clinical Manifestations of CAN are numerous. Some are minor, such as resting tachycardia, exercise intolerance, orthostatic tachycardia and bradycardia syndromes. Some others may lead to complications, including intraoperative and perioperative cardiovascular instability, orthostatic hypotension, silent myocardial ischemia, or autonomic cardiopathy associated with left ventricular diastolic dysfunction [5]. Ewing et al. [6] proposed 5 tests in 1985 to diagnose CAN in diabetic patients.

We assumed that altered response to hypoxemia due to CAN and non-functional central neurological breathing command could play a key role in these sudden deaths, and this assumption is the base of this brief report on sudden serious cardiorespiratory events without any associated clinical signs in 2 type 1 diabetic patients with severe CAN hospitalized in our department.

## Case presentation

Ewing’s tests were used to diagnose CAN in our 2 patients. Heart rate responses to deep breathing and to standing up were measured using an electrocardiogram. Blood pressure response to standing up was measured automatically by Dinamap®. We couldn't perform the Valsalva test because of proliferative retinopathy presence in the 2 patients.

The first patient was a 54-year-old woman, on insulin therapy since discovery of type 1 diabetes in 2000 in context of polyuria-polydipsia syndrome. Since then, her diabetes was poorly controlled, with multiple hospitalizations for ketoacidosis and chronically elevated HbA1c level between 11 and 15% (97 and 140 mmol/mol respectively). Diabetes complications therefore developed, including laser-required diabetic retinopathy and end stage chronic kidney disease requiring dialysis. However, this patient had not experienced any history of impaired cardiac or respiratory condition. She was non-smoking and her last cardiovascular evaluation in 2015 reported no evidence of supra aortic trunks atheroma and a low coronary calcium score of 14.

The second patient was a 55-year-old type 1 diabetic woman. Her diabetes was discovered in 1973 in relation to a ketoacid coma. Insulin therapy was immediately started. As her diabetes began early in childhood and was poorly controlled, she also developed a severe retinopathy that is treated, as well as an end-stage kidney failure that has required kidney transplantation. Many cardiovascular comorbidities were present, including ischemic cardiomyopathy with left ventricular dysfunction, multiple vascular lower limb stenting and bypass, hypertension and bilateral transtibial amputation. In contrast, there was no impaired respiratory condition detected so far.

Both patients were admitted in the podiatry care unit of the Diabetology Department at the Pitié-Salpêtrière Hospital (Paris, France) due to an infected lower limb wound, respectively located on the left heel and the right lower limb stump.

Like every new admitted patient, they were screened for CAN, confirmed in both cases to be severe, as per Ewing’s classification. Indeed, they both displayed an orthostatic hypotension associated to an inappropriate heart rate response to standing up and to deep breathing.

The common denominator of these 2 clinical cases was isolated low blood saturation on pulse oximeter during the systematic nurse check-up (77% and 85% respectively) contrasting with the absence of any complaint such as dyspnea, polypnea or signs of respiratory insufficiency observed during the clinical examination (Table 1). Of note, arterial blood gas measurements subsequently confirmed that blood oxygen saturation was low and that both patients were indeed hypoxemic.

- Patient 1. Since few days before the acute event, no efficient ultrafiltration was performed because of patient drowsiness. The patient presented with vascular overload with lower limbs edema, pleural effusion and several hypoxemia episodes objectified by a daily pulse oximetry around 90–92% without oxygen therapy contrasting with the absence of dyspnea or signs of respiratory failure. Close monitoring of oxygen saturation was performed. Few minutes after the observation of 77% saturation, on the pulse oximeter, confirmed by blood gas measurements (PaO_2_ 59 mmHg − PaCO_2_ 55 mmHg), the patient was found unconscious, without any breathing and cardiac pulse (absence of carotid and femoral pulses and absence of pulse recorded by plethysmography). She instantly had chest compressions and endotracheal intubation for artificial ventilation. After 2 min of cardiopulmonary resuscitation, she recovered spontaneous cardiac rhythm without any cardiac electric shock or drugs. She was transferred to the intensive care unit. A thoracic CT scan eliminated a pulmonary embolism but reported abundant bilateral pleural effusion. Unchanged ECG, normal echocardiography and negative troponin were strong arguments against myocardial infarction. Fever and blood inflammation’s markers were absent, so was evidence of metabolic disorders like dyskalemia or hypoglycemia. Cardiac arrest was considered to be probably caused by hypoxemia in light of the quick recovery observed after ventilation, as well as blood gas analysis reporting hypoxemia and hypercapnia. As no drugs with central respiratory depressant effect had been given the previous days, the main retained risk factor of hypoxemia was vascular overload due to insufficient dialysis volume removal. Continuation of dialysis sessions allowed for progressive volume depletion (decreased weight from 75.7 to 60.8 kg) and correction of hypoxemia with PaO2 ≥ 90 mmHg on blood gas analysis.
- Patient 2. During the systematic nurse check-up, low oxygen saturation at 85% on the pulse oximeter was recorded. There was no dyspnea nor respiratory discomfort. An oxygen mask at 9L/min was then administered allowing a 95% pulse oxymetry recovery. She was then transferred to the cardiac intensive care unit, following discovery of pulmonary edema with pleural effusion detected by pulmonary examination and chest CT scan. In view of increased troponin, modified ECG with ST depression and T wave inversion in the inferolateral territory and cardiac hypokinesia during echocardiography, a diagnosis of pulmonary edema secondary to acute coronary syndrome was retained. As coronarography showed no progression of the coronary lesions, no revascularization procedure was undertaken. The patient recovered normal oxygen blood saturation after diuretic medication combined with non invasive ventilation and evacuating pleural punction. As left ventricular ejection fraction was found at 33% on echocardiography, β blockers were introduced and increased gradually.

**Table 1:** Patients CAN features and clinical respiratory outcomes during the episode

	Patient 1	Patient 2	Normal
*CAN*			
HR to deep breathing	1.05	1.01	> 1.11
HR to standing up	1.0	1.09	> 1.12
BP drop to standing up	+	+	−
*Acute hypoxemia*			
SaO2 (%)	77	85	> 95%
PaO2 (mmHg)	59	60	83–108
PaCO2 (mmHg)	55	37	32–45
Breath rate (/min)	12	14	12–20
Dyspnea	−	−	
Labored breathing	−	−	
Paradoxical breathing	−	−	
Cyanosis	+	−	

*CAN* cardiac autonomic neuropathy, *HR* heart ratio, *BP* blood pressure, *PaO2* Partial pressure of oxygen in arterial blood, *PaCO2* Partial pressure of carbon dioxide in arterial blood, *SaO2* Oxygen saturation on pulse oximeter

## Discussion and conclusion

The common denominator of the 2 cases is the absence of dyspnea or precursory signs of the sudden cardiorespiratory event contrasting with the presence of confirmed hypoxemia by pulse oximeter plus blood markers, and the presence of an evident hypoxemia etiology.

Furthermore, in both cases, such absence of precursory signs seemed to be related to the severity of the diabetic neurological autonomic disorder which could be objectified by CAN screening. That is why impaired hypoxic ventilatory drive to hypoxemia induced by diabetic autonomic neuropathy is highly suspected to cause sudden cardiac deaths. Our observations are in agreement with previously published reports of unexplained sudden deaths in patients with CAN. A recent meta-analysis of 14 studies involving a total of 5647 SCD cases and 346,356 participants showed a two-fold higher risk of SCD in subjects with diabetes [7]. Presence of CAN was found to be a significant contributor to such observations. Indeed, in an analysis of 2900 diabetic subjects, Vinik et al. showed that the subset of those with CAN (defined by an abnormal Ewing’s test) had a significant 2.14 relative risk of death, even escalating up to 3.65 if CAN was defined by the presence of more than 2 abnormal quantitative autonomic function tests [5], thereby showing a clear relationship between CAN severity and mortality. Several 90′s studies previously brought to light dysfunction of the central neurological breathing command in patients with CAN leading to hypoxemia. For example, central obstructive sleep apnea [8] and desaturation episodes under 85% [9] were shown to be more prevalent in diabetic patients with CAN than in those without. Furthermore, impaired hypoxic ventilatory drive in diabetic patients with CAN was also underlined by numerous studies [10, 11]. In fact, compared to control participants, basal baroreflex sensitivity is blunted in type 1 diabetic patients with CAN. These latter are not able to spontaneously increase their ventilation to fight against hypoxia, and short-term oxygen administration could restore temporarily the baroreflex sensitivity [12].

All those results constitute a solid body of evidence suggesting that patients with CAN lose their ability to increase minute ventilation to hypoxia. Physiopathologically, chemosensors of ventilatory drive are no more stimulated by hypoxemia. Since chemosensors such as carotid bodies, sensitive to arterial oxygen pressure, are innerved by parasympathetic system, patients displaying a severe CAN probably present an impaired hypoxic ventilatory drive response by impairment of their autonomic nervous system. Delayed hypoxemia management due to absence of warning clinical signs could favor occurrence of sudden cardiorespiratory events and lead to dramatic consequences such as death. Diabetic heart is typically accompanied by a deeper grade of myocardial tissue hypoxia. As a marker of hypoxia, carbonic anhydrase enzyme expression is increased in human diabetic ischemic cardiomyopathy and mediates endothelial cells and myocytes death [13]. These observations highlight the potential role of carbonic anhydrase in the events triggered by the dysregulation favored by CAN.

To conclude, the important point is that hypoxemia can be easily missed and the diagnosis of cardio-pulmonary disease delayed since no clinical signs of respiratory failure are reported in these two clinical cases. Systematic screening of CAN and proactive detection of impaired hypoxic ventilatory drive for early management (e.g. treatment of hypoxemia) should be systematically undertaken in diabetic patients to prevent its dramatic consequences such as cardiorespiratory arrest and death. This would also allow for careful handling of respiratory depressant medication in this population at higher risk of SCD, particularly in case of associated cardiac and pulmonary chronic diseases, general anesthesia, severe sleep apnea syndrome or any medical situation exacerbating hypoxemia. Co-morbidities presented by our 2 patients represent limitation of out report. Not only hypoxemia but other function in respiratory physiology might also be contributing factors to unfavourable outcomes [14] such as cardiorespiratory arrest.

## Data Availability

Data are available on request from the corresponding author.

## Abbreviations

BP: Blood pressure
CAN: Cardiac autonomic neuropathy
DCCT: Diabetes Control and Complications Trial
HR: Heart ratio
PaO2: Partial pressure of oxygen in arterial blood
PaCO2: Partial pressure of carbon dioxide in arterial blood
SaO2: Oxygen saturation on pulse oximeter
SCD: Sudden cardiac deaths
